# Molecular Identification and Analysis on Differential Pathogenicity of *Cylindrocarpon* Species Associated With Ginseng Root Rust Rot in Northeastern China

**DOI:** 10.3389/fpls.2022.894104

**Published:** 2022-06-28

**Authors:** Zibo Li, Chunlei Sun, Xinran Liu, Rujun Zhou

**Affiliations:** Department of Plant Protection, Shenyang Agricultural University, Shenyang, China

**Keywords:** ginseng root rust rot, causal agent, identification, pathogenicity difference, *Cylindrocarpon*

## Abstract

*Panax ginseng* C. A. Meyer is one of the most important medicinal herbs in China. It is known for its high medicinal value and economic value. The ginseng root rust rot (RRR) has always been one of the important diseases troubling the ginseng industry. The yield reduction rate of RRR is ~30%. To understand why the *Cylindrocarpon* species bring about the ginseng RRR in Northeastern China, this study isolates 45 strains from samples collected in Liaoning, Jilin, and Heilongjiang provinces. The rDNA-internal transcribed spacer (ITS) sequence was analyzed to identify the pathogenic species. The morphological characteristics of colonies and conidia of each strain on potato dextrose agar (PDA) medium were observed, and the pathogenicity difference between different pathogenic species was analyzed by pricking method and determining the cell wall degrading enzyme activity. The BLAST alignment analysis shows that the homology of rDNA-ITS between 45 strains and *Cylindrocarpon* is more than 99%, among which 28 are identified as *Cylindrocarpon destructans*, three are identified as *C. destructans* var. *destructans*, and 14 are identified as *Ilyonectria robusta*. The colony diameters of all 45 isolated range from 4.21 ± 0.16a to 7.78 ± 0.25c cm after several days of incubation. Among all the species, *I. robusta* has the fastest growth rate, and *C. destructans* var. *destructans* has the slowest growth rate. Pathogenicity test results show that the pathogenicity of *C. destructans* var. *destructans* is the strongest, followed by *C. destructans*. *I. robusta* has relatively weak pathogenicity.

## Introduction

*Panax ginseng* Meyer (Araliaceae) is a perennial medicinal plant of Acanthopanax Familia (Wang, 2001), and it is featured by high medicinal and economic values. The major production regions of ginseng are Liaoning, Jilin, and Heilongjiang provinces in Northeastern China. In recent years, with the restructuring of agroecological planting, more efforts have been put into the protection of forest resources. The mode of cultivated ginseng on the flat farmland has been strengthened and has gradually replaced the traditional mode of woodland planting ginseng. This transformation marks an important development direction of the ginseng industry in China. The ginseng RRR, caused by *Cylindrocarpon destructans* (Zinns.), is one of the main root diseases in the flat farmland. With ginseng aging and more serious incidences, this disease reduces the yield and commodity value of ginseng directly. The ginseng RRR has restricted the development of the Chinese ginseng industry over the past years (Bai, 1990). It can infect the main root, lateral root, fibrous root, and rhizome of the ginseng. The suffering plant has the following symptom: the upper part is short and the leaves turn yellow or red. The roots of ginseng turn red, rust-colored, oval, and irregular in shape. The disease spots are mixed together, and the ginseng epidermis is damaged when the disease became serious. In the final stage of disease, the pathogen goes deep into the root tissue, causing the root to be desiccated or even die. According to statistics, the incidence of RRR could even reach up to 80% in the serious ginseng-producing areas in China (Fu, 2007). Due to the latent infection (Song et al., 2014), ginseng RRR can survive for a long time in the soil or overwinter in the disease residue as an initial source of infection in the following year (Kim et al., 2009). Moreover, the disease occurs more seriously in continuously cropping fields of ginseng.

Based on the traditional morphological analysis, Yan (2002) classified 12 strains of rust rot pathogen isolated from Changbai Mountain into four groups with different pathogenicity, namely *C. destructans, Cylindrocarpon panacis, Cylindrocarpon obtusisporum*, and *Cylindrocarpon panicicola*. *Cylindrocarpon destructans* and *C. panacis* are more pathogenic, while *C. obtusisporum* and *Cylindrocarpon panicicol* are relatively weak. However, the fungi in the asexual stage are morphologically similar and difficult to identify. The rDNA-internal transcribed spacer (ITS) has been widely used in the intraspecific taxonomy of fungi due to its abundant informative sites and conservative sequence length (Seifert et al., 2004). Song et al. (2014) identified 15 strains of rust rot pathogen from different regions in Korea using rDNA-ITS and mt SSU rDNA sequence analysis and classified them into two different groups according to the pathogenicity. Cell wall degrading enzyme (CWDE) is an important pathogenic factor in the infection of host plants by pathogens (Ilona et al., 2005). It facilitates host plant invasion and destruction by enzymatically hydrolyzing plant cell wall structures and producing nutrients required for pathogen growth (Kian et al., 2009). In recent years, the reports on ginseng RRR mainly focused on the morphological identification (Natasha et al., 2020), biological analysis (Li et al., 2020), and pesticide screening of the pathogens (Zhang et al., 2020), while the studies of ginseng RRR species and pathogenicity by molecular technology were still poorly understood.

In this study, 45 strains of rust rot pathogens were isolated from the major ginseng agricultural regions by rDNA-ITS sequence analysis based on the existing fungal taxonomy (Crous et al., 2006), and the differences in morphology and pathogenicity among each species were analyzed. In this way, this study aimed to provide a theoretical basis for the comprehensive control of ginseng RRR.

## Materials and Methods

### Materials

Of note, 3-year-old ginseng seedlings (mean height 10.23 cm and 3 branches) were used for testing. A total of 45 strains of rust rot pathogens were isolated from different regions, such as Liaoning Province, Jilin Province, and Heilongjiang Province (Table 1). The tested medium used was potato dextrose agar (PDA) (200 g potato, 17 g agar, 20 g glucose, and 1,000 ml distilled water). The reagents and instruments include a new plant genomic DNA extraction kit (Tiangen, Beijing), DNA marker-C (Shenggong, Shanghai), 2 × Es Taq Master Mix (Dye) (Kangwei, Beijing), general electrophoresis instrument (ABI, USA), gel imaging analysis system (UVP, USA), biomicroscopy (NIKON, Beijing), acetic acid-sodium acetate buffer (50 mmol/L, pH 5.5), citric acid-sodium citrate buffer (50 mmol/L, pH 5.0), and 0.1 mol Na-acetate buffer (pH 5.0, containing 1 mol·L^−1^ NaCl and 1 mmol·L^−1^ EDTA). The universal primers used were ITS1 (5′-TCCGTAGGTGAAC-CTGCGG-3′) and ITS4 (5′-TCCTCCGCTTATTGATAT-GC-3′) [Shenggong, Shanghai PCR instrument (ABI, USA)].

**Table 1:** Collecting location and strain information of ginseng root rust rot (RRR).

**Site of collection**	**Code of strains**	**Isolates number**
Yulin Town Ji'an City, Jilin Province	Yl036, YL026	2
Songjiang Town, Baishan City, Jilin Province	FS027, FS028	2
Tai Town, Ji'an City, Jilin Province	JA030, JA031	2
Xijiang Town, Tonghua City, Jilin Province	TH029, TH002	2
Taiwang Town, Ji'an City, Jilin Province	YC022, YC024, YC025	3
Hulin County, Hulin City, Heilongjiang Province	HL023	1
Qinghe County, Harbin City, Heilongjiang Province	QH043	1
Hongsheng Town, Fushun City, Liaoning Province	XB004, XB040, XB008, XB006, XB048, XB047, XB050, XB007, XB042, XB049, XB051, XB011, XB009, XB052, XB046	15
Taiping Town, Dandong City, Liaoning Province	KD018, KD020, KD019, KD014, KD016	5
Hualai Town, Benxi City, Liaoning Province	HR001, HR002	2
Dasu River Town, Fushun City, Liaoning Province	R13, R7, R5-4, R5-3	4
	X5, X7, X25-5, X25-4, X25-1, X25-3	6

### Methods

#### Isolation of Ginseng RRR

The modified Zhongda's (Wan, 2007) tissue separation method was adopted, and the epidermis of 3 ×3 mm at the junction of disease and healthy was disinfected with 75% alcohol. Then, the tissues were sterilized in 20% sodium hypochlorite solution for 30 s to 2 min, washed with sterile water three times, and placed on a PDA medium. Colonies grew after 3–5 days under the dark culture at 20°C. The marginal hyphae were picked up on PDA medium under the pure culture at 20°C.

#### rDNA-ITS Sequence Analysis of Ginseng RRR

For 45 strains of the pathogen, the mycelial plugs with a diameter of 5 mm were taken from the edge of the pathogen colony, then cultured for 15 days, and finally cultured in the dark on a PDA medium for 15 days at 20°C. The DNA of the pathogen was extracted using the kit extraction method and then stored at 20°C. The universal primers, namely, ITS1 (5′-TCCGTAGGTGAAC-CTGCGG-3′) and ITS4 (5′-TCCTCCGCTTATTGATAT-GC-3′) were selected to amplify the fungi by PCR. The design of the reaction system and procedure referred to the method of Fu et al. (2012). PCR products were sequenced by ShengGong Bioengineering Service Co., Ltd., (Shanghai, China), the sequencing results were compared and analyzed by BLAST in GenBank (http://www.ncbi.nlm.nih.gov), and the phylogenetic tree was built using the adjacency method with the MEGA6.0 software.

#### Determination of Growth Rate and Observation of Spore Morphology

Mycelial plugs of 5 mm diameter were taken from the edge of the pathogen colonies, cultured for 15 days, and finally cultured on the PDA medium in the dark at 20°C for 11 days. The colony diameter was measured using the cross method and repeated three times. A small amount of sterile water was dropped onto the center of the glass slide. A minimal hypha was picked out in the center of the colony with a clean silver. The glass slide was covered for a while, then the spore morphology was observed, and taken pictures under a 40 × lens using a NIKON biomicroscope.

#### Determination of Pathogenicity

Ginseng roots were inoculated *in vitro* by needling. The surface of 3-year-old ginseng roots was disinfected with 75% ethanol, and 5 points were stabbed with a clean dissection on each tested plant. Each wound was ~0.25 mm in diameter and 1.00 mm in depth (Rahman and Punja, 2006). The spore suspensions of ginseng RRR were dripped to the wound with a concentration of 1 ×10^6^, and the aseptic water treatment was used as a control. After inoculation, the infected ginsengs were placed in a large Petri dish with a humidity of 95–100% at 20°C for observation. Treatments were performed on five healthy ginseng plants and repeated three times. The incidence rates of the disease were investigated 28 days later. The criteria of disease severity index referred to the methods of Rahman and Punja (2005): 1 = no obvious damage, 2 = brown spot with ~0.9 mm diameter, 3 = dark brown spot with ~1–1.4 mm diameter, 4 = black spot with ~4–7.0 mm diameter, 5 = black spot > 7.0 mm diameter, and the disease spots connected with each other, 6 = whole plant infection. The calculation formula is as follows: disease severity index (DSI) = ([X1 ×1] + [X2 ×2] + [X3 ×3] + [X4 ×4] + [X5 ×5] + [X6 ×6])/(X1 + X2 + X3 + X4 + X5 + X6), where X1, X2, X3, X4, X5, and X6 represent the number of plants with rotting severity of 1, 2, 3, 4, 5, and 6, respectively.

Three representative strains of ginseng RRR were selected for testing, namely, *C. destructans* (X25-5), *I. robusta* (YL026), and *C. destructans* var. *destructans* (R7). Polygalacturonase (PG) substrate: 10 g·L^−1^ polygalacturonic acid solution. Polymethylgalacturonase (PMG) substrate: 10 g·L^−1^ pectin solution. Carboxymethyl (Cx) substrate: 10 g·L^−1^ sodium methylcellulose (CMC) solution. β-glucose lichen substrate: 10 g L^−1^ salicylate solution. These substrates were stored at 4°C. Galacturonic acid standard curve and glucose standard curve were drawn. According to different reaction substrates, the activities of PG, PMG, and Cx were determined using the 3–5 dinitrosalicylic acid (DNS) method. Units of enzyme activity: the amount of enzyme needed to catalyze the substrate to produce 1 μ mol galactose acid per minute at 37°C was expressed by U/ml or U/mg.

### Data Analysis

The experimental data were statistically analyzed using the SPSS 19.0 software, and the significance of differences was tested using the Duncan's method.

## Results and Analysis

### Isolation of Ginseng RRR

A total of 45 strains were isolated from the major ginseng production regions in Northeastern China (Table 1). Each strain was named according to the collection site.

### ITS Sequence Analysis on *C. destructans*

**Figure 1:**
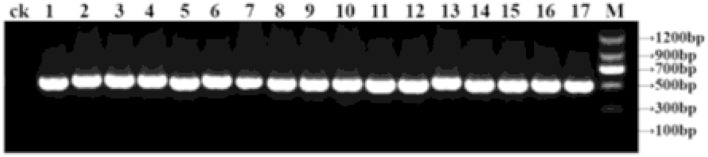
PCR amplification of rDNA-internal transcribed spacer (ITS) from part of isolates. M: Marker; 1-17: bacterial strain XB4, XB7, XB52, X7, YL26, XB34, TH002, HR002, HL23, R13, KD018, X5, XB050, XB007, XB042, XB049, and XB05; ck: ddH_2_O.

**Figure 2:**
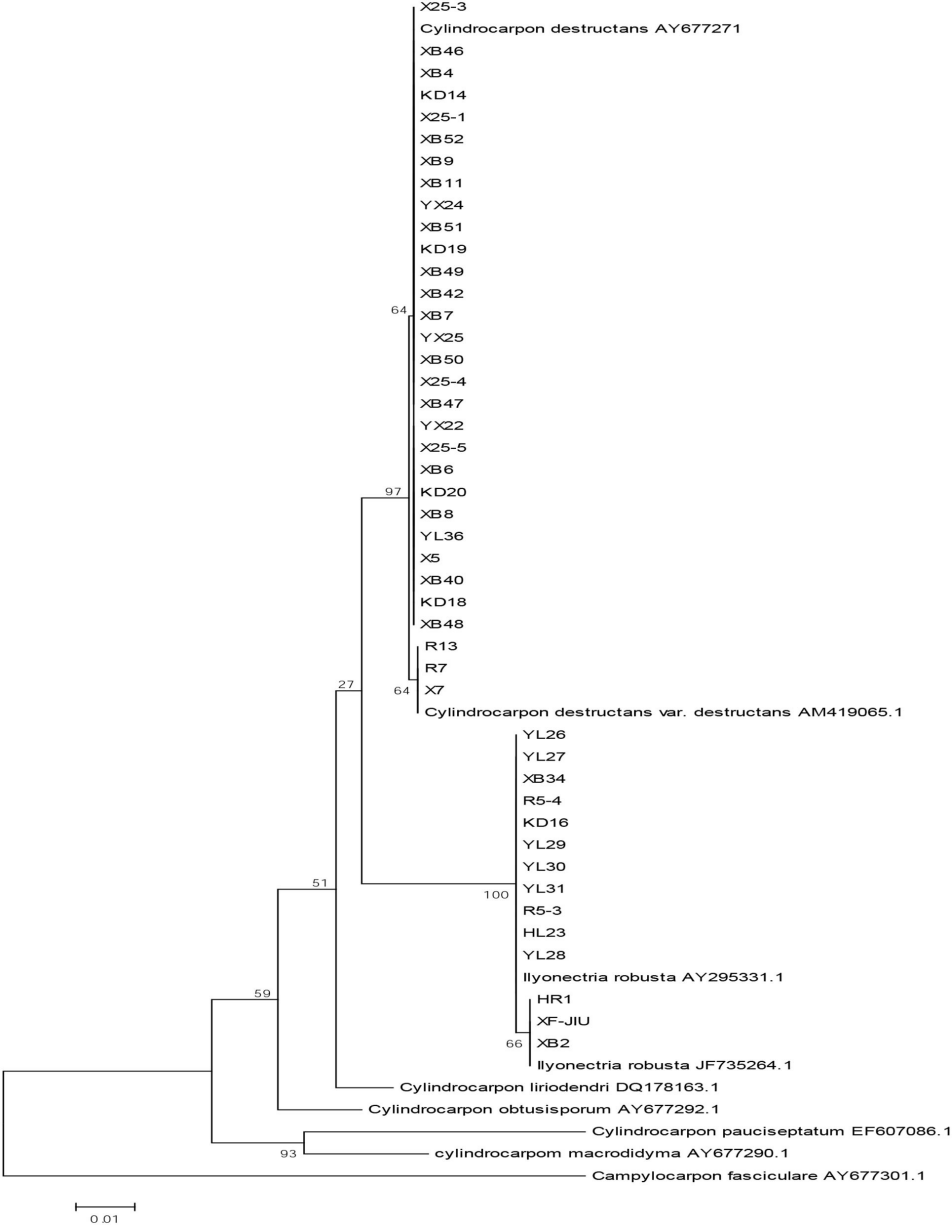
The phylogenetic tree of *Cylindrocarpon* and other related isolates based on rDNA-ITS sequence (using adjacency method using the MEGA 6.0 software).

The ITS1/ITS4 primers were used for PCR amplification of 45 strains. Nucleotide sequences of ~500 bp were obtained by 1% agarose gel electrophoresis (Figure 1). In the phylogenetic analysis, the sequences of the tested strains were compared with the known species in GenBank. The sequences with high homology were selected and added to *Gampylocarpon fascicular*, which was used as the external reference sequence (Samuels and Brayford, 1990; Halleen et al., 2004; Abreo et al., 2010; Tewoldemedhin et al., 2011; Cabral et al., 2012). M A phylogenetic tree was built using the MEGA 6.0 software. In addition, adjacent clustering analysis was performed in Figure 2. BLAST analysis showed that the sequence of rDNA-ITS was 99% similar to the *Cylindrocarpon* in GenBank. As shown in Figure 2 and Table 2, the tested strains could be divided into three species, namely, *C. destructans* var. *destructans* (3 strains), *C. destructans* (28 strains), and *I. robusta* (14 strains).

**Table 2:** Classification of 45 *Cylindrocarpon* isolates with rDNA-internal transcribed spacer (ITS) sequence analysis.

**Group**	**Species**	**Total number**	**Code of strains**
1	*Cylindrocarpon destructans*	28	XB046, KD014, X25-1, XB052, XB09, XB011, YC024, XB051, KD019, XB049, XB042, XB007, YC025, XB050, X25-4, XB047, XB048, KD018, XB040, X5, YL036, XB008, KD020, XB006, X25-5, YC022, XB004, X25-3
2	*Cylindrocarpon destructans* var. *destructans*	3	R13, R7, X7
3	*Ilyonectria robusta*	14	YL026, FS027, QH043, R5-4, KD016, TH029, JA030, JA031, R5-3, HL023, FS028, HR001, HR002, TH002

### Morphological Observation of *C. destructans*

**Figure 3:**
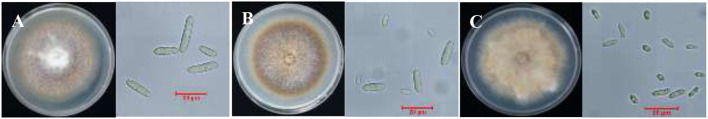
Typical colonies and macroconidia of *Cylindrocarpon* isolated from ginseng root rust rot. **(A)** *Cylindrocarpon destructans*; **(B)** *C. destructans* var. *destructans*; **(C)** *Ilyonectria robusta*.

On PDA medium, most colonies of the 45 strains were white or beige at the beginning and then they gradually turned brown or dark brown. The colony edges were neat. Most of the colonies were prostrate in shape. The aerial mycelia were luxuriant. Compared with the other species, the *C. destructans* var. *destructans* was the darkest in color—dark brown, while the *I. robusta* was the lightest in color—brownish gray. There were significant differences in growth rates among the different strains. After 11 days, the diameter of 45 strains was 4.21–7.78 cm. Among them, strain R5-4 had the fastest growth rate with a colony diameter of 7.78 cm, while strain R13 had the slowest growth rate with a colony diameter of 4.21 cm. The large conidia were visible, cylindrical, straight, or curved with one or more septa. Both ends of the large conidia were obtuse. The small conidia differed from the large conidia. Most of the shapes of small conidia were elliptic and did not have septa. The differences in colony morphology, conidia morphology, and growth rate among the different species are shown in Figure 3 and Table 3.

**Table 3:** Morphological data and pathogenicity test of *Cylindrocarpon* isolated from ginseng RRR.

**Group**	**Species**	**Colonies appearance**	**Colony diameter (cm)**	**Spore morphology**	**Disease severity index**	**Pathogenicity**
1	*Cylindrocarpon destructans*	Light brown, pilotaxitic	5.26c	Cylindrical, erect, slightly narrow stem	3.20 b	++
2	*Cylindrocarpon destructans* var. *destructans*	Dark brown, fluffy	4.52b	Clavate, curved, obtuse stem	3.47 a	+++
3	*Ilyonectria robusta*	Gray, flocculent	6.68a	Long elliptic, slightly curved, obtuse at both ends	2.87 c	+

+++ *Indicates strong pathogenicity and can cause typical pound rot symptoms*.

++ *Means strong pathogenicity, which can cause rust decay symptoms or tissue decay*.

+ *Indicates general pathogenicity, causing inoculation point*.

*Adverse symptoms of mild rust or tissue decay were shown nearby (Yan, 2002)*.

### Pathogenicity Analysis of *C. destructans*

**Figure 4:**
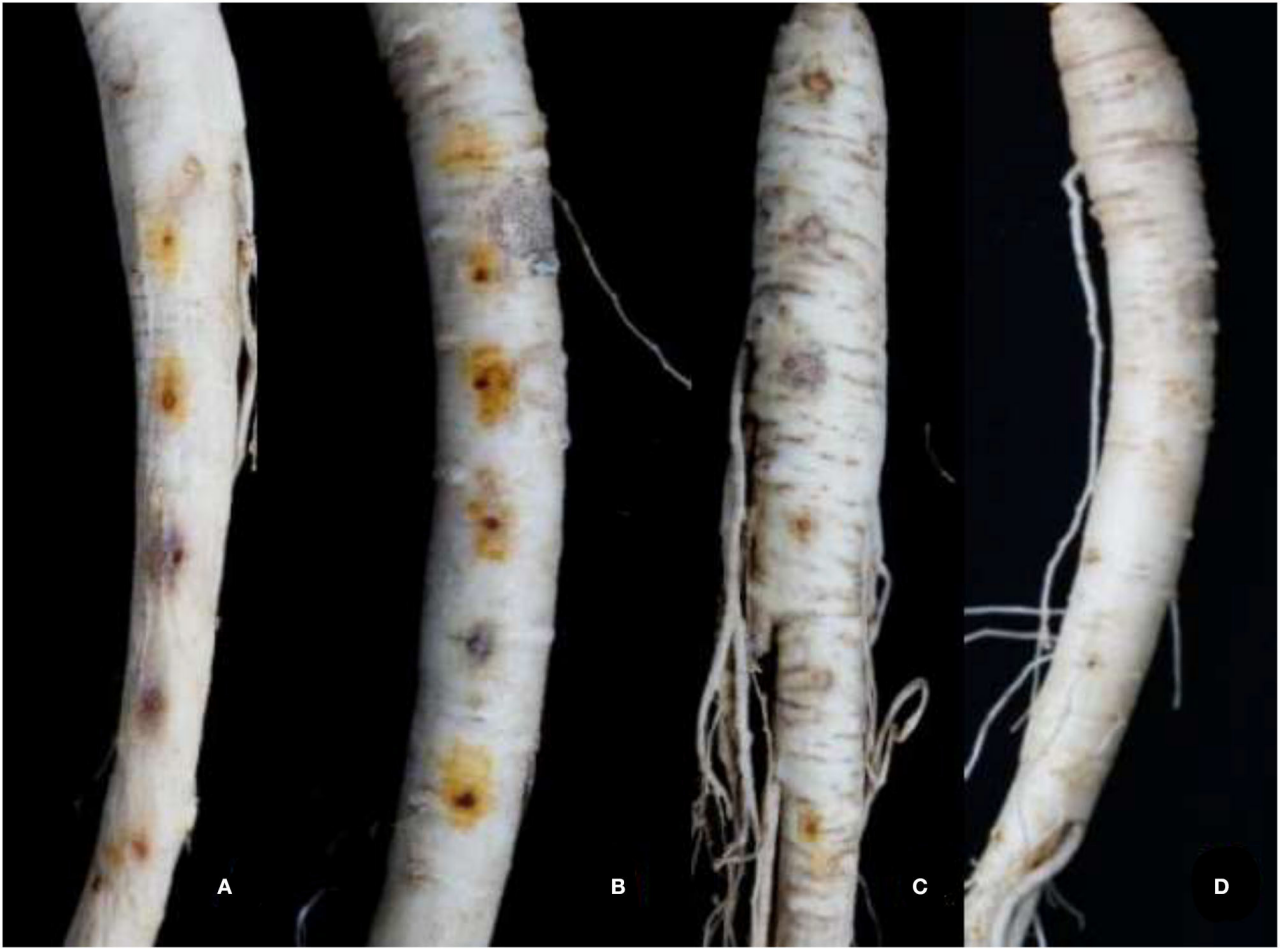
The symptoms of ginseng root based on the different species of *Cylindrocarpon* infected. **(A)** *C. destructans*; **(B)** *C. destructans* var. *destructans*; **(C)** *I. robusta*; **(D)** Control treatment.

The strains are inoculated *in vitro* based on different groups. Different groups show different pathogenicity, which indicates the differences in pathogenicity between species. Among them, *C. destructans* var. *destructans* has the strongest pathogenicity, and the yellow rust spot becomes obvious after inoculation of ginseng for 2 days. Second, *C. destructans* has small rust spots after inoculation of ginseng for 3 days. *Ilyonectria robusta* has relatively weak pathogenicity. The tested ginseng shows yellow rust spots and slight rots after inoculation of ginseng for 5 days. With the passing of time, yellow rust spots and slight decay appear on the tested ginseng. The spots of *C. destructans* var. *destructans* are the most serious, while other species show different pathogenicity (Figure 4).

### CWDE Activity

**Figure 5:**
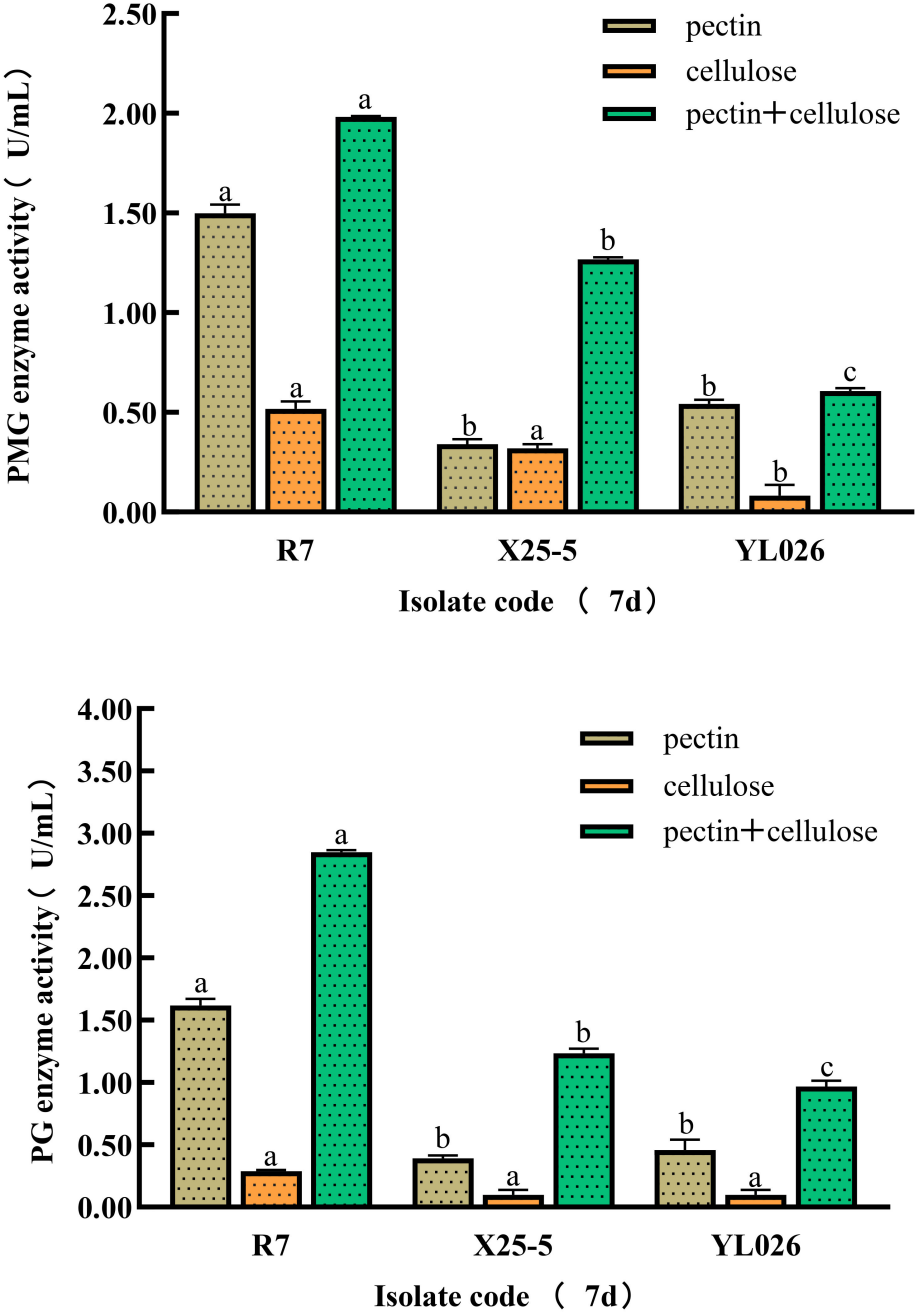
Polymethylgalacturonase (PMG) and polygalacturonase (PG) activity in different kinds of ginseng RRR pathogen. Different letters in each column indicate statistically significant differences at the 0.05 probability level according to the Duncan test. a,b,c indicates the statistical difference without any specific meaning.

Figure 5 shows that all three kinds of pathogenic strains can induce the production of PMG and PG in different culture media. The mixed culture medium of pectin and cellulose is the most effective in inducing the production of PMG and PG, and the strain R7 is the most capable of producing PMG and PG. The enzyme activity from high to low in order is: R7 > X25-5 > YL026.

**Figure 6:**
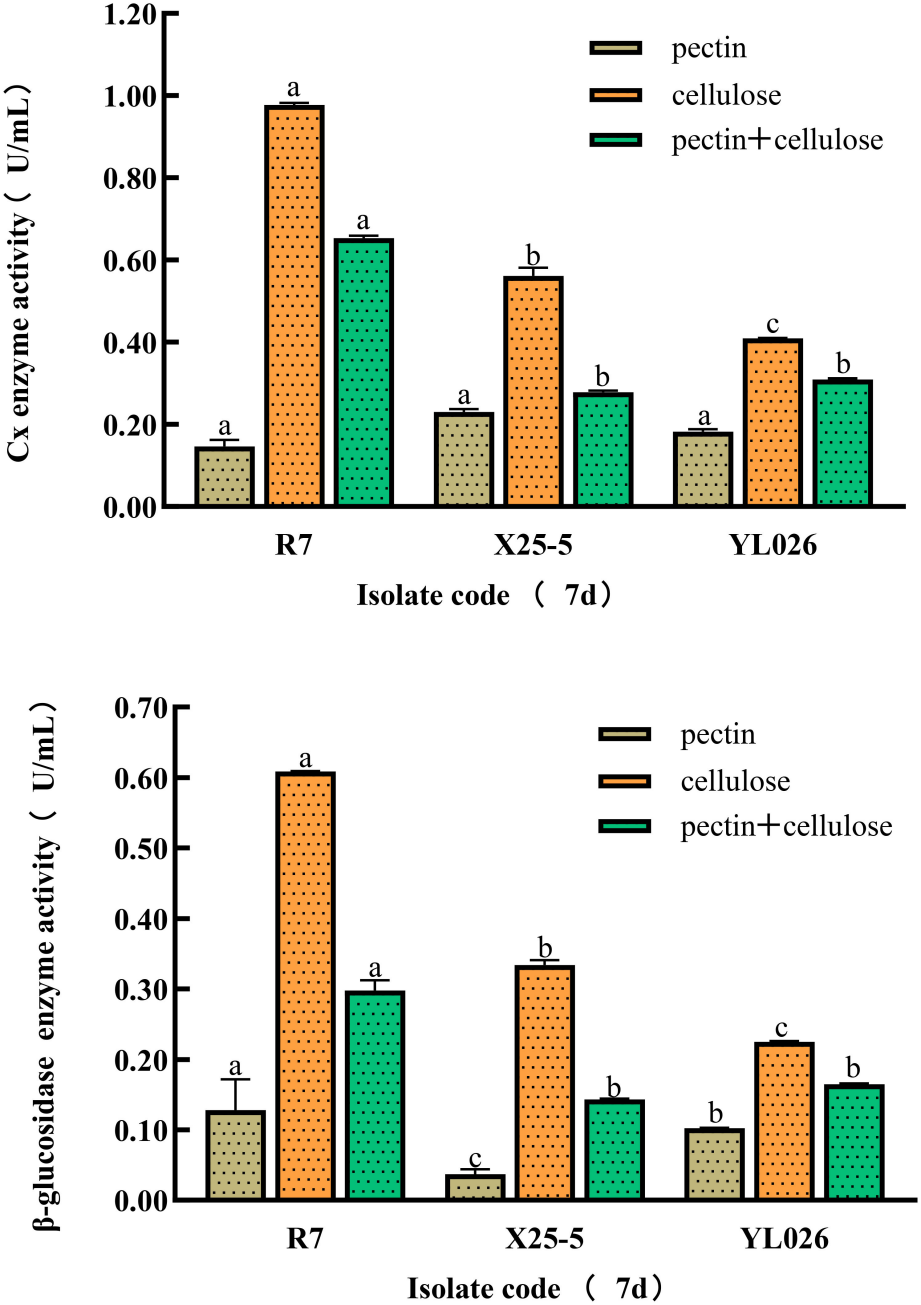
Carboxymethyl (Cx) and β-glucosidase activities in different kinds of ginseng RRR pathogen. Different letters in each column indicate statistically significant differences at the 0.05 probability level according to the Duncan test. a,b,c indicates the statistical difference without any specific meaning.

Figure 6 shows that all the pathogenic strains can induce the production of Cx and B-glucose in different culture media. The cellulose culture medium performs the best for the production of Cx and B-glucose, and strain R7 is the most capable of producing Cx and B-glucose. The enzyme activity from high to low in order is: R7 > x25-5 > YL026.

## Discussion

Ginseng RRR was first identified by American plant pathologist Van Hook in the investigation of American ginseng diseases in 1904. Qi was the first to report the ginseng RRR in China back in 1966, and now ginseng RRR has become one of the most serious problems hindering ginseng production. At present, seven species of pathogenic fungi of ginseng RRR have been reported in China (i.e., *C. destructans, C. panacis, C. obtusisporum, C. panicicola, C. destructans* var. *destructans, Cylindrocarpon didymum*, and *Cylindrocarpon vaginae*) (Yan, 2002). In addition, due to the wide range of hosts, the *Cylindrocarpon* infects not only the medicinal plants but also the fruit trees (Cabral et al., 2012). Yared (Tewoldemedhin et al., 2011) isolated four species of *Cylindrocarpon* from the apple rust roots in 2011. The *C. destructans* caused the most serious damage to the roots of apple trees. Based on the above studies, the strains of *Cylindrocarpon* were comprehensively compared in this experiment. The gene sequences suitable for constructing a phylogenetic tree were downloaded and screened out from the Genbank database. It was also compared with the tested strains to accurately identify the pathogens. Among them, the pathogen *I. robusta* mentioned in this study was initially isolated from the American ginseng in Canada (Hildebrand, 1935). These species can cause RRR on various plants, such as American ginseng, grape, and aconite (Wang et al., 2015). In 2015, Lu et al. first reported the ginseng RRR symptoms caused by *I. robusta* in Jilin Province (Lu et al., 2015). *Ilyonectria robusta* can be proven to be one of the pathogenic fungi of ginseng RRR. The *I. robusta* isolated and identified in this experiment showed 99% homology with the strains of *I. robusta* reported by Lu after rDNA-ITS sequence analysis. The colony morphology and anamorph were basically consistent with the study by Lu. In addition, most strains of the *I. robusta* in this study were isolated from Jilin Province, the same region as the *I. robusta* strains reported in Lu's study. However, most strains of *C. destructans* and *C. destructans* var. *destructans* were isolated from Liaoning Province. It is speculated that the distribution of pathogenic species may be geographically related.

The genus of *Cylindrocarpon* was first named by Wollenweber with the sexual stage of *Neonectria* (Farh et al., 2017). However, the existing taxonomic studies have shown that *Neonectria/Cylindrocarpon* is a composite species group (Mantiri et al., 2001). Chaverri et al. (2011) classified the *Neonectria* into four genera, namely, *Ilyonectria, Neonectria* (asexual *Cylindrocarpon*), *Rugonectria*, and *Thelonectri* based on the phylogenetic analysis and the morphology differences. Among them, the *Nectria radicicola* (*C. destructans* perfect stage) leading to ginseng RRR were named *Ilyonectria*.

Rahman and Punja (2005) showed significant differences between the strong pathogenic and weak pathogenic strains in morphology. Similar results were also found in this study. After *C. destructans* var. *destructans* was cultured on a PDA medium for 2 weeks, the colonies were light brown at first and then turned dark brown. The disease spots were large and expanded rapidly with the strongest pathogenicity in inoculation. The *I. robusta* was yellow in the same culture condition and turned brown-gray in the later period. The pathogenicity of *I. robusta* was weaker than the other two strains. In this study, the activity of CWDEs of *C. destructans* var. *destructans* was measured. According to the test results, the activity of CWDEs of *C. destructans* var. *destructans* was higher than the other two species. It is indicated that the activity of CWDEs produced by strong pathogenic species was higher than the weak pathogenic species. This result is consistent with the study by Cano-Canchola et al. (2000).

It can be seen that *I. robusta* was distributed in Liaoning, Jilin, and Heilongjiang provinces; *C. destructans* was distributed in Liaoning and Jilin provinces; and *C. destructans* var. *destructans* was detected only in Qingyuan County, Fushun City, and Liaoning Province, as shown in Figure 2. According to the rDNA-ITS sequence analysis, *I. robusta* was widely distributed in the three provinces, but the genetic distance is 0.001 ± 0.001. It indicates that there are differences in *I. robusta* strains among different provinces, and these differences are caused by different geographical environments in genetic evolution. *Cylindrocarpon destructans* was distributed closely in Liaoning and Jilin provinces, and the genetic distance is 0.000 ± 0.000. We speculated that population exchange may exist in *C. destructans*. *Cylindrocarpon destructans* var. *destructans* was detected only in Liaoning Province, and all three species were detected in the same investigation site. We speculated that using fungicides for a long time may lead to the variation of *Cylindrocarpon*.

Due to the limited number of isolated pathogenic strains, only preliminary studies on the taxonomy (Gao et al., 2016) and identification and pathogenicity differences of ginseng RRR pathogens could be conducted. To control the ginseng RRR, the intraspecific relationships of the pathogens and the interaction mechanisms with rhizosphere soil and host plants of ginseng should be analyzed in future studies.

## Conclusion

In this study, 45 strains of ginseng RRR pathogen were isolated and purified from the disease samples collected along with the ranges of Changbai Mountain in Liaoning, Jilin, and Heilongjiang provinces. All the strains were classified into three species based on rDNA-ITS sequencing analysis, including *C. destructans, C. destructans* var. *destructans*, and *I. robusta*. It is confirmed that there are significant differences in the colony and criminal morphology, growth rate, and pathogenicity among the three species.

## Data Availability Statement

The original contributions presented in the study are included in the article/supplementary material, further inquiries can be directed to the corresponding author.

## Author Contributions

Conceptualization: ZL and XL. Methodology, formal analysis, and investigation: ZL and CS. Writing—original draft preparation: ZL. Writing—review and editing: RZ. All authors contributed to the article and approved the submitted version.

## Funding

This study was supported by the key project at Central government level. The ability establishment of sustainable use for valuable Chinese medicine resources (No. 2060302).

## Conflict of Interest

The authors declare that the research was conducted in the absence of any commercial or financial relationships that could be construed as a potential conflict of interest.

## Publisher's Note

All claims expressed in this article are solely those of the authors and do not necessarily represent those of their affiliated organizations, or those of the publisher, the editors and the reviewers. Any product that may be evaluated in this article, or claim that may be made by its manufacturer, is not guaranteed or endorsed by the publisher.
